# *In silico* Logistic Model for Table Olive Related Microorganisms As a Function of Sodium Metabisulphite, Cinnamaldehyde, pH, and Type of Acidifying Agent

**DOI:** 10.3389/fmicb.2016.01370

**Published:** 2016-08-31

**Authors:** Verónica Romero-Gil, Antonio Garrido-Fernández, Francisco N. Arroyo-López

**Affiliations:** ^1^Regulatory Council of PDO Aloreña de Málaga Table OlivesMalaga, Spain; ^2^Food Biotechnology Department, Instituto de la Grasa (CSIC), Campus Universitario Pablo de OlavideSeville, Spain

**Keywords:** olive packaging, predictive microbiology, preservatives, sulphites, cinnamaldehyde

## Abstract

A probabilistic/logistic model, based on binary data (growth/no growth), was used to assess the effects of sodium metabisulphite (SM) and cinnamaldehyde (CIN; 0–1000 mg/L) against the main microbial groups found in table olive environment [lactic acid bacteria (LAB), yeasts, and *Enterobacteriaceae*], according to pH (range 3.5–5.0), and type of acidifying agent (HCl or pyruvic acid). The inhibitory effect of SM depended on the pH while that of CIN was scarcely influenced by it (except for LAB). LAB were more sensitive to SM, while yeasts were to CIN. The use of pyruvic acid for correction of pH always produced a reduction (compared to HCl) of the inhibitory power of both preservatives. The *in silico* models for HCl showed that, at pH 4.0, and growth probability 0.01, the LAB population might be inhibited by the presence in the medium of 150 mg/L SM or 1000 mg/L CIN, while in the case of yeasts, 450 mg/L SM, or 150 mg/L CIN are required. No growth of *Enterobacteriaceae* was observed at this (or lower) pH level. The results obtained may contribute to the stabilization of non-thermally treated table olive packaging.

## Introduction

The olive tree is well adapted to the Mediterranean climate and its products (table olives and olive oil) are basic elements of the culture and diet of many countries (Spain, Turkey, Egypt, Greek, or Italy) around the basin. Nowadays, the worldwide production of table olives exceeds 2.6 million tons/year according to the last final balance for the crop year 2013–2014 (IOOC, 2015), with green Spanish-style (olives debittered by alkaline treatment), natural (directly brined) olives, and Californian style (olives darkened by oxidation in an alkaline medium) as the main preparation types (Garrido-Fernández et al., 1997). Uncontrolled growth of microorganisms during olive packaging may cause product spoilage due to the production of CO_2_, swollen containers, softening of fruits, clouding of brines, or consumption of lactic acid in aerobic conditions. Hence, the microbiological stabilization of the final products during the commercialization period is critical, and there is a need to improve the knowledge of the factors required to achieve it (Arroyo-López et al., 2009).

Due to its high pH (close to neutrality), ripe olive packaging requires sterilization while Spanish-style, and natural olives are fermented products that may be preserved by different methods (modified atmosphere, vacuum, use of preservatives, pasteurization, or by their physicochemical characteristics) (Garrido-Fernández et al., 1997). Nowadays, the thermal treatment is widely applied; however, it may cause undesirable changes in the traditional flavor of several presentations, particularly seasoned fruits which should, then, be stabilized by preservatives (Arroyo-López et al., 2009). Currently, the preservatives and the levels permitted for addition in table olives vary according to legislations. In the Trade Standards Applying to Table Olives (IOOC, 2004), benzoic, and sorbic acids (or their respective salts) are the only preservatives permitted at maximum doses of 1000 mg/kg (benzoic) and 500 mg/kg (sorbic acid) or 1000 mg/kg for their combination. In the EU, both preservatives are also allowed but at levels of 1000 mg/kg (sorbic) and 500 mg/kg (benzoic), expressed as acids (Regulation EU 1129/2011). Finally, the CODEX ^1^Standard for Table Olives (STAN 66-1981 rev 2013), and CODE^2^X Standard for Food Additives (STAN 192-1995 rev 2014) permit the addition of higher levels of benzoic (2000 mg/kg) and sorbic (1000 mg/kg) acids, as well as the use of sulphites (metabisulphite, sulfur dioxide, or bisulphite) at a maximum dose of 100 mg/kg flesh in the final product. Hence, there are evident discrepancies between the EU legislation and CODEX concerning the levels and preservatives allowed in table olives. Such differences may lead to disputes and insecurity in the international table olive commercial trading. Therefore, studies on the inhibitory effects of preservatives on table olive related microorganisms are necessary to assist legislators on the homogenization of standards.

The use of sorbic and benzoic acids in table olive packaging could also have some drawbacks. Among the most important are: (i) accumulation in the olive (flesh) fat, with the subsequent limitation of their effects in the brines, (ii) development of undesirable sensorial notes for consumers, (iii) browning of fruits, and (iv) degradation by microorganisms (Garrido-Fernández et al., 1997; Arroyo-López et al., 2005). As a result, the table olive sector is demanding research for obtaining more appropriate preservatives. Particularly, sulphites, which, in addition to their proved antimicrobial activity, may also produce an important, and persistent antioxidant effect for preventing browning (Arroyo-López et al., 2008; Echevarria et al., 2010; Segovia-Bravo et al., 2010). In this content, the contribution of Juneja and Friedman (2008) on the effect of carvacrol and cinnamaldehyde (CIN) for facilitating the thermal destruction of *Escherichia coli* O157:H7 in raw ground beef, and the results obtained by Taboada-Rodríguez et al. (2013) on sodium metabisulphite (SM), potassium sorbate, and dimethyl dicarbonate are particularly pertinent.

In a previous study, the individual inhibitory effects of diverse preservatives on lactic acid bacteria (LAB) and yeasts cocktails isolated from table olives, at fixed levels of pH (4.0) and salt (5%), were tested *in vitro* using a dose-response model (Romero-Gil et al., 2016). Among the diverse compounds assayed, SM, CIN, and pyruvic acid (PYR) showed the higher perspective of application in table olives. The present work represents a further step in this research, using a probabilistic/logistic model to determine the influence of pH and type of acidifying agent (HCl or PYR) on the inhibitory effects of SM and CIN, also expanding the work to the *Enterobacteriaceae* population. Predictive microbiology is a useful tool to describe the response of microorganisms as a function of environmental variables quantitatively (McMeekin et al., 1993). By using this mathematical approach, it is possible to determine: (i) the more sensitive microbial gropup to preservative and type of acidifying agent, (ii) the compound with the highest inhibitory effects on microorganisms, and (iii) the growth/no growth (G/NG) boundaries of microorganisms as a function of the preservative, pH levels, and type of acidifying agent.

## Materials and methods

### Microorganisms and cocktail preparation

A total of 24 strains belonging to different LAB, yeasts, and *Enterobacteriaceae* species were used in the present study. Many of the LAB and yeast strains were previously isolated from diverse table olive trade preparations and identified by molecular methods (data not shown). Many of them belong to the Table Olive Microorganisms Collection (TOMC) of Instituto de la Grasa (CSIC, Seville), while the *Enterobacteriaceae* strains were kindly supplied by Dr. Antonio Valero Díaz (University of Córdoba, Spain), and purchased from the CECT (Spanish Type Culture Collection, University of Valencia, Spain). Their references and origin are shown in Table 1. Inoculum were prepared by introducing one single colony of each strain into 5 ml of a YM broth medium (Difco™, Becton, and Dickinson Company, Sparks, USA) for yeasts, 5 ml of a MRS broth medium (de Man, Rogosa, and Sharpe; Oxoid, Cambridge, UK) for LAB, or 5 ml of VRBD (Crystal-violet Neutral-Red bile glucose) broth medium (Merck, Darmstadt, Germany) for *Enterobacteriaceae*. After 48 h of incubation at 30°C, 1 ml from each tube was centrifuged at 9000 × *g* for 10 min, the pellets were washed with sterile saline solution (9 g/L), centrifuged and re-suspended again in 0.5 mL of a sterile saline solution to obtain a concentration of about 7 log_10_ CFU/mL for yeasts and 8 log_10_ CFU/mL in the case of LAB and *Enterobacteriaceae*, which were confirmed by enumeration on appropriate media. The microorganism suspensions belonging to each group were gently mixed in the same proportions, obtaining three different cocktails (yeast, LAB, and *Enterobacteriaceae*), which were then used to inoculate the media described below.

**Table 1:** **Microbial strains used in the present study for preparation of the LAB, yeasts, and ***Enterobacteriaceae*** cocktails**.

**Microbial group**	**Species**	**Strain**	**Origin**
LAB	*Lactobacillus pentosus*	TOMC-LAB2	Spanish-style green olive fermentations Gordal variety (Spain)
		TOMC-LAB3	Spanish-style green olive fermentations Gordal variety (Spain)
		TOMC-LAB4	Spanish-style green olive fermentations Hojiblanca variety (Spain)
		TOMC-LAB5	Spanish-style green olive fermentations Gordal variety (Spain)
		TOMC-LAB6	Spanish-style green olive fermentations Manzanilla variety (Spain)
	*Lactobacillus plantarum*	NC8	Grass silage (Norway)
		TOMC-LAB9	Directly brined olive fermentations Gordal variety (Spain)
	*Lactobacillus paraplantarum*	TOMC-LAB12	Green Spanish-style olive fermentations (Spain)
	*Pediococcus pentosaceus*	P56	Fermented food. University of Valencia (Spain)
		FBB-63	Fermented food. Michigan State University (United States)
Yeast	*Candida diddensiae*	TOMC-Y1	Spoilage of directly brined green olive packaging (Spain)
	*Issatchenkia occidentalis*	TOMC-Y3	Spoilage of directly brined green olives packaging (Spain)
	*Saccharomyces cerevisiae*	TOMC-Y4	Spoilage of directly brined green olives packaging (Spain)
	*Debaryomyces hansenii*	TOMC-Y25	Directly brined green olive fermentations Manzanilla-Aloreña variety (Spain)
	*Pichia membranifaciens*	TOMC-Y31	Directly brined green olive fermentations Manzanilla-Aloreña variety (Spain)
	*Candida boidinii*	TOMC-Y47	Spoilage of directly brined green olive packaging (Spain)
	*Candida tropicalis*	TOMC-Y72	Spoilage of directly brined green olive packaging (Spain)
	*Lodderomyces elongisporus*	TOMC-Y73	Spoilage of directly brined green olive packaging (Spain)
*Enterobacteriaceae*	*Escherichia coli*	CECT 405	American Cyanamid Co. (United States)
		CECT 4267^*^	Human feces, stool from outbreak of hemorrhagic colitis (United States)
		CECT 4782^*^	Human stool from outbreak of hemorrhagic colitis (United States)
	*Salmonella enterica* subsp. *enterica*	CECT 443	Food poisoning (United Kingdom)
		CECT 556	Water, Albufera lake (Spain)
		CECT 4396	Human gastroenteritis (Denmark)

* *E. coli serotype O157:H7*.

*Excluding the Enterobacteriaceae strains, all microorganisms were also previously used by Romero-Gil et al. (2016) for testing the inhibitory effects of diverse preservatives by using a dose-response model*.

### Growth media and data collection

Sterilized YM, MRS, or VRBD broth were modified with 5% NaCl and adjusted to different pH levels (3.5, 4.0, 4.5, and 5.0) by HCl (37% purity, Applichem Panreac, Damstadt, Germany), or PYR (99% purity, Merck, Damstadt, Germany) additions. The three basal media were then individually supplemented with SM (Applichem Panreac) and trans-CIN (Sigma-Aldrich, St Luis, USA) at 0, 25, 50, 100, 250, 500, and 1000 mg/L. The experimental design consisted in a full-factorial design with 56 different treatments for SM and other 56 for CIN (7 levels for preservative^*^4 levels of pH^*^2 types of acidifying agents) with 7 replicates by treatment in the case of LAB and yeasts. For *Enterobacteriaceae*, 7 replicates were performed for experiments with HCl, while 3 replicates were carried out in the case of PYR. The design was individually executed for each microbial cocktail, making a total of 2128 treatments.

Growth was monitored in a Bioscreen C automated spectrophotometer (Labsystem, Helsinki, Finland) with a wideband filter (420–580 nm). Measurements were taken every 2 h after a pre-shaking of 5 s for 7 days, making a total of 178,752 raw data to be analyzed. The wells of the microplate were filled with 20 μL of inoculum and 330 μL of medium (according to treatment as described above), always reaching an initial OD of approximately 0.2 (inoculum level above 6 log_10_ CFU/mL). The inocula were always above the detection limit of the apparatus, which was determined by comparison with a previously established calibration curve. Uninoculated wells for each experimental series were also included in the microplate to determine, and subsequently, subtract the noise signal. For each well, growth (coded as 1) was assumed when the OD increase on the initial OD (after subtraction of the noise signal) was higher than 0.1; no-growth (coded as 0) was recorded when the initial OD remained stable or the increase was <0.1. Thus, only a binary data (0 or 1) is possible for each assay. Responses from each replicate were recorded independently, and the whole matrix was subjected to statistical analysis. After concluding the experiments, randomly selected wells (which included both growth and no-growth samples, representing 1% of the total cases) were spread on YM, MRS, or VRBD agar plates and their counts were estimated to corroborate G/NG assumption (data not shown).

### The *in silico* probabilistic/logistic model

A logistic regression model links the probability of occurrence of a conditional event (Y), which depends on a vector (x) of explanatory variables. The quantity, p(x) = E (Y/x) represents the conditional mean of y (in this case growth probability) given x (environmental factors) when the logistic distribution is used. In its simplest form, it takes the expression:

$$\begin{array}{l} \text{logit }\left(\text{p}\right)\;=\;\ln\left[\text{p}\left(\text{x}\right)/\left(1-\text{p}\left(\text{x}\right)\right)\right]\;=\beta_{0}+\ldots +\beta_{\text{n}}{\text{x}}_{\text{n}} \end{array}$$

where β_0_+ ⋯ +β_i_ are the intercept and the coefficients of the polynomial function and x_i_ (*i* = 1…n) are the environmental variables.

The predicted survival probability as a function of the independent variables, deduced from the logistic regression, is as follows:

$$\begin{array}{l} \text{p }\left({\text{x}}_{\text{i}}\ldots {\text{x}}_{\text{n}}\right)\;=\;\exp\left(\text{logit }\left(\text{p}\right)\right)/\left(1+\exp\left(\text{logit }\left(\text{p}\right)\right)\right) \end{array}$$

According to the number of independent variables, two dimension probability curves (given particular values of another variables), and the G/NG interfaces for selected probabilities can be deduced. The logistic regression model was fit to the binary data (G/NG) obtained from the different treatments (combinations of type of preservative, pH, acidifying agent, and microbial cocktail), using XLSTAT software package (2015.4.01.20116, Addinsoft, Paris, France). The initial model assumed was:

$$\begin{array}{l} \text{logit }\left(\hat{p}\right)\;=\text{ Intercept}+\text{pH}+\left[\text{P}\right]+\text{A}+\text{pH}\cdot \left[\text{P}\right]+\text{pH}\cdot \text{A}\\ \;+\left[\text{P}\right]\cdot \text{A}+\text{pH}\cdot \left[\text{P}\right]\cdot \text{A} \end{array}$$

where pH stands for the −log_10_[H+] in the medium, P for the different preservatives assayed (SM and CIN), and A for the type of acidifying agent (HCl or PYR). For building the equations, the model using HCl for the pH correction was the reference, and its equation just consisted of the sum of all the terms (preserving their signs) which does not include A-PYR. The model for treatments with pH corrected with PYR was built by adding to the previous ones the terms including A-PYR (but without this indication, which was used just as a label).

For the selection of variables, the stepwise backward option, with 0.05, and 0.10 *p*-values to enter and remove, respectively, was used. The number of maximum allowable runs was set to 100 and the tolerance to 0.0001. Validation was achieved with a total of 575 cases (275 for SM and 300 for CIN), randomly selected and not used for the model building (XLSTAT software).

The log-likelihood statistic was used to evaluate the significant contribution of each term in the model to the response (G/NG data). To verify the overall fit, the McFadden, the Cox and Snell, and the Nagelkerke R-squares were used. The null hypothesis was tested by −2log(likelihood), Score, and Wald and Hosmer-Lemeshow statistics. Also, overall hit rate, sensitivity, and sensibility were also estimated (Hosmer and Lemeshow, 2000).

The interpretation of the model is usually based on the multiplicative form of logit (*p*) which establishes that changes in the odds ratio are expressed by exp(b), which means the change in the odds ratio caused by one unit change in the variable of interest when the others remain at the same level. A value of 1 (*e*^0^ = 1) indicates that the variable under study does not cause any effect on the odds ratio, values <1 reduce it while values >1 increase it (Hosmer and Lemeshow, 2000). The predicted growth probabilities for the preservative concentration, pH, and acidifying agent combinations according to the microbial cocktail, and their corresponding G/NG boundaries for diverse probabilities (*p* = 0.15, 0.10, 0.05, and 0.01) were deduced from the expression ln[p/(1 − p)] = logit(p).

## Results

### Logistic models for the lab cocktail

A total of 559 cases were used to build the model for LAB (292 for SM and 267 for CIN), with a distribution of G/NG data of 169/123 for SM, and 209/58 for CIN. The probabilistic model for both preservatives fit the data satisfactorily as demonstrated by values obtained from the diverse tests (see Table S1 in Supplementary Material). The goodness of fit was also assessed by the overall hit (accuracy) to the data used in model development and validation, which indicate an almost perfect segregation between G/NG treatments for both SM and CIN (Table S2, Supplementary Material). The specificity (true no-growth rate) were 97 (SM) and 98% (CIN) while the sensitivity (true growth rate) were 96 (SM) and 100% (CIN). Furthermore, the predictions obtained for the 225 validation cases (100 for SM and 125 for CIN) also led to high values of specificity (90 for SM and 95% for CIN) and sensitivity (97 for SM and 97% for CIN). Therefore, it can be stated that LAB models achieved an adequate segregation between G/NG data and can be considered appropriate for representing the G/NG events of this microbial group as a function of the levels of SM, CIN, pH, and type of acidifying agent.

For both preservatives and types of acids used for pH correction, the models for the logit (*p*) of LAB population can be easily deduced from the coefficients (Table S3, Supplementary Material). These equations were:

$$\begin{array}{l} \text{For SM}-\text{HCl}:\text{ logit}\left(\text{p}\right)\;=-9.983+3.024^{*}\text{pH}-0.077^{*}\left[\text{SM}\right]\\ \;+0.011^{*}{\text{pH}}^{*}\left[\text{SM}\right]\\ \text{For SM}-\text{PYR}:\text{ logit}\left(\text{p}\right)=-64.880+17.290^{*}\text{pH}+0.024^{*}\\ \;\left[\text{SM}\right]-0.006^{*}{\text{pH}}^{*}\left[\text{SM}\right]\\ \text{For CIN}-\text{HCl}:\text{ logit}\left(\text{p}\right)=24.325-4.305^{*}\text{pH}\\ \;-0.125^{*}\left[\text{CIN}\right]+0.029^{*}{\text{pH}}^{*}\left[\text{CIN}\right]\\ \text{For CIN}-\text{PYR}:\text{ logit}\left(\text{p}\right)=-59.190+16.199^{*}\text{pH}+0.019^{*}\\ \;\left[\text{CIN}\right]-0.007^{*}{\text{pH}}^{*}\left[\text{CIN}\right] \end{array}$$

**Figure 1:**
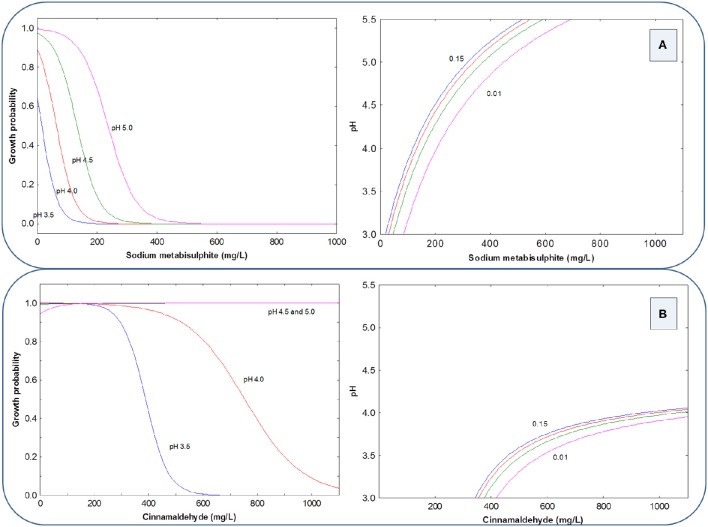
**Growth probability and G/NG interfaces of the LAB cocktail as a function of SM (A) and CIN (B) concentrations in a pH range from 3.5 to 5.0, using HCl as an acidifying agent**.

The growth probability (y-axis) of the LAB cocktail in the presence of SM or CIN, in a 2D graph, can be deduced by using the preservative concentrations as the x-axis and assigning selected values to pH (5.0, 4.5, 4.0, and 3.5). Using HCl as an acidifying agent, the growth probability graph shows a strong inhibitory effect of SM on LAB growth, and also that the inhibition increases as the pH levels decrease (curves shifted to left; Figure 1A left). This way, at pH 5.0, 500 mg/L of SM inhibited LAB cocktail but, at pH 3.5, the concentration required was around 150 mg/L. The CIN had, in general, a lower inhibitory power that SM for this microbial group, and was also markedly influenced by pH (Figure 1B left); in fact, LAB were able to grow at any CIN concentrations at pH 5.0 and 4.5, and only were inhibited at pH 4.0 or lower. At pH 3.5 the inhibitory effect of CIN began at 200 mg/L, but was necessary 600 mg/L of CIN to achieve a complete inhibition of the LAB cocktail.

Also, by plotting pH levels *vs*. preservative concentrations, the G/NG interfaces at different growth probabilities (0.15, 0.10, 0.05, and 0.01) can be visualized, and the graph is particularly useful for selecting the appropriate combination to achieve inhibition at a predetermined probability. The LAB G/NG interfaces for SM and CIN, using HCl as acidifying agent, shows the considerable effect of the correction of pH with HCl on the inhibitory power of both preservatives, especially on CIN (curves with a reduced inhibition region, always below pH 4.0; Figures 1A,B, right). Thus, at pH 4.0 (usual pH value in olive packaging conditions) and 0.01 growth probability (or 0.99 inhibition), LAB control requires about 150 mg/L of SM but around 1000 mg/L of CIN. In general, the G/NG interfaces are useful to deduce the different combinations of preservatives, and pH levels that potentially can control the growth of the LAB cocktail at selected growth probabilities, depending on the risk to be assumed by the operator.

**Figure 2:**
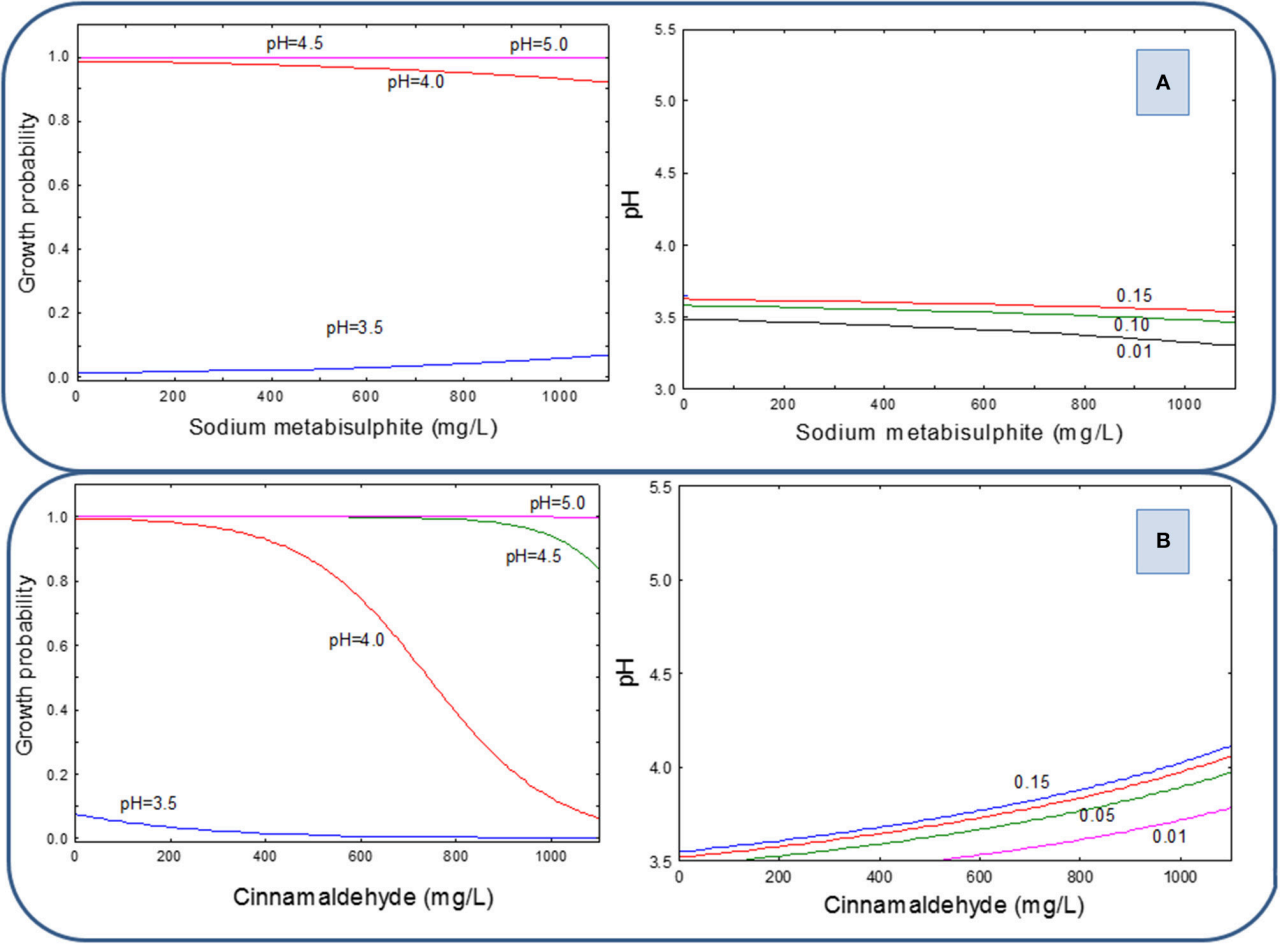
**Growth probability and G/NG interfaces of the LAB cocktail as a function of SM (A) and CIN (B) concentrations in a pH range from 3.5 to 5.0, using PYR as an acidifying agent**.

When the LAB cocktail was inhibited with the same preservatives but using PYR for the pH correction, the 2D graphical representation of growth probability showed that regardless of the type of preservative assayed, growth was prevented only at pH below 3.5 (Figures 2A,B, left). The same conclusion is deduced from the respective G/NG interfaces (Figures 2A,B, right). Then, the pH correction with PYR markedly reduces the inhibitory effects of both SM and CIN since the inhibition observed at pH = 3.5 may be due just to the extremely low pH value reached.

### Logistic models for the yeast cocktail

For this eukaryotic microorganisms, 584 cases were analyzed, 292 for SM and other 292 for CIN, with a distribution of G/NG data of 238/54 for SM, and 156/136 for CIN. The probabilistic models for both preservatives fit the experimental data satisfactorily, as upheld by the diverse statistical tests (Table S1 in Supplementary Material). The goodness of fit was also assessed by the overall hit (accuracy) to the data used in model development and validation, which indicate an almost perfect segregation between G/NG treatments (Table S2 in Supplementary Material). The specificity was 96 (SM) and 98% (CIN) while the sensitivity was 100 (SM) and 97% (CIN). Furthermore, the predictions obtained for 200 validation cases (100 for SM and other 100 for CIN) also led to high specificity (100 for SM, 98% for CIN) and sensibility (99 for SM, 98% for CIN). Therefore, it can be stated that models achieved an adequate segregation between G/NG data and can be considered appropriate for representing the G/NG yeast events as a function of the levels of SM, CIN, pH, and type of acidifying agent.

The models for logit (p) of yeasts for both preservatives can be deduced from the respective coefficients (Table S4 in Supplementary Material) as already explained for LAB. The equations for the models, according to preservative and acid used for the pH correction were:

$$\begin{array}{l} \text{For SM}-\text{HCl}:\text{ logit}(\text{p})\;=-26.199+8.915^{*}\text{pH}-0.063^{*}\\ \;[\text{SM}]+0.009^{*}{\text{pH}}^{*}[\text{SM}]\\ \text{For SM}-\text{PYR}:\text{ logit}(\text{p})\;=23.446-3.417^{*}\text{pH}-0.039^{*}\\ \;[\text{SM}]+0.006^{*}{\text{pH}}^{*}[\text{SM}]\\ \text{For CIN}-\text{HCl}:\text{ logit}(\text{p})\;=4.507+0.431^{*}\text{pH}-0.062^{*}\\ \;[\text{CIN}]-0.001^{*}{\text{pH}}^{*}[\text{CIN}]\\ \text{For CIN}-\text{PYR}:\text{ logit}(\text{p})=9.130-0.496^{*}\text{pH}-0.050^{*}\\ \;[\text{CIN}]+0.003^{*}{\text{pH}}^{*}[\text{CIN}] \end{array}$$

**Figure 3:**
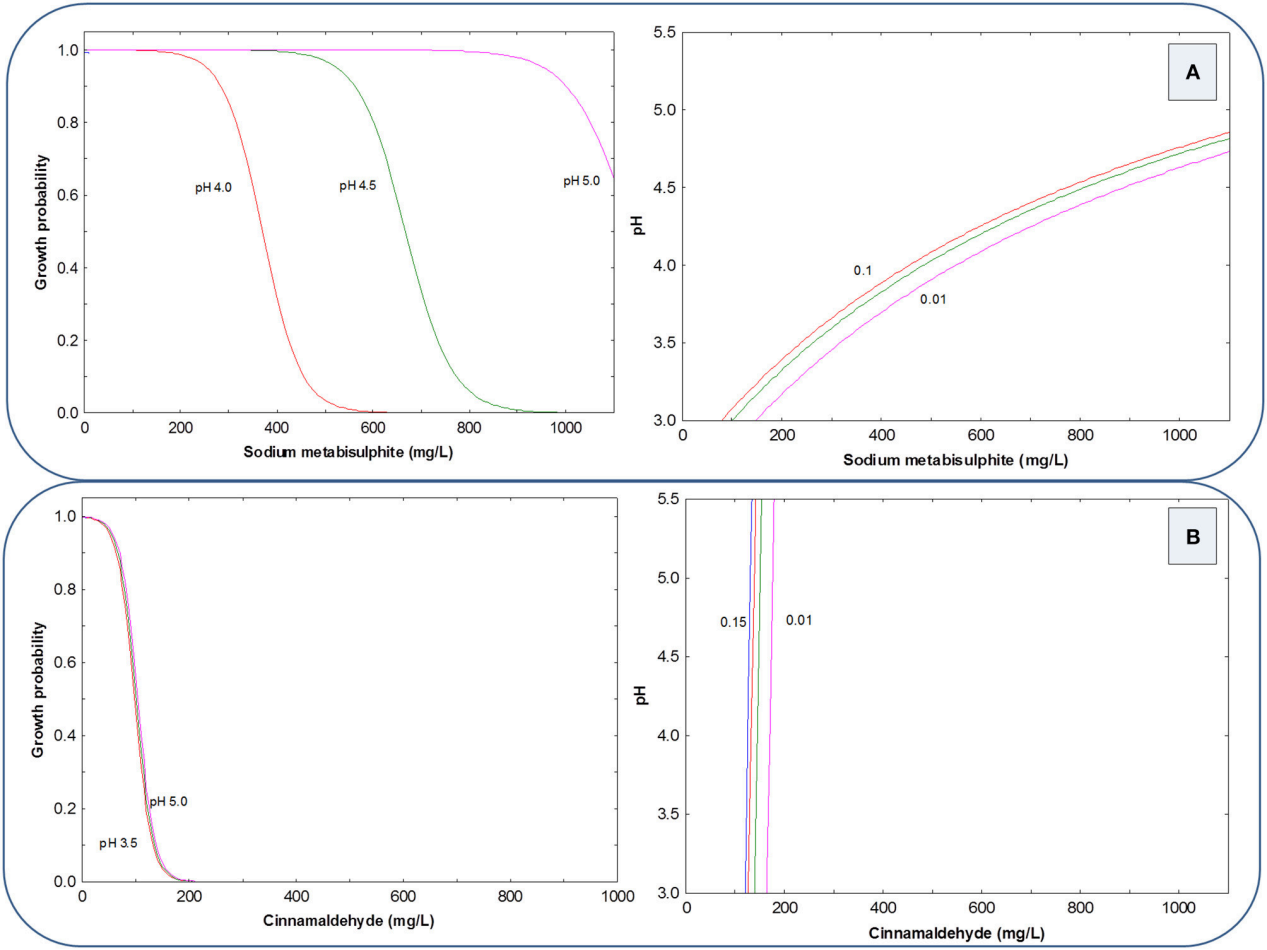
**Growth probability and G/NG interfaces of the yeast cocktail as a function of SM (A) and CIN (B) concentrations in a pH range from 3.5 to 5.0, using HCl as acidifying agent**.

As in LAB, the graphical representation of these equations shows that for HCl, the inhibitory effect of SM against the yeast cocktail was strongly dependent on the pH levels (Figure 3A, left). In fact, at pH 5.0, there was always (within the range of concentrations assayed) yeast growth, and at pH 4.5, the growth probability decrease begins around 400 mg/L and reaches the total inhibition at above 900 mg/L. Interestingly, at pH 4.0, the inhibition begins at 200 mg/L and the growth was totally inhibited at approximately 600 mg/L SM (Figure 3A left). On the contrary, there was not observed any influence of pH on the inhibitory power of CIN. In fact, the curves for pH from 3.5 to 5.0 overlapped. Therefore, the total inhibition of the yeast cocktail can be achieved, independently of the pH values, at (or above) 200 mg/L of CIN (Figure 3B left). The G/NG interfaces for the yeast cocktail at different growth probabilities (0.15, 0.10, 0.05, and 0.01) show the above-mention interactions in the complete experimental region (ranges of both preservative and pH). The effect of pH is only observed on SM. At high pH are required high concentrations of SM to control the yeast cocktail (Figure 3A right) while, on the contrary, the G/NG interfaces for CIN are pH independent, and practically perpendicular lines to the pH-axis (Figure 3B right).

**Figure 4:**
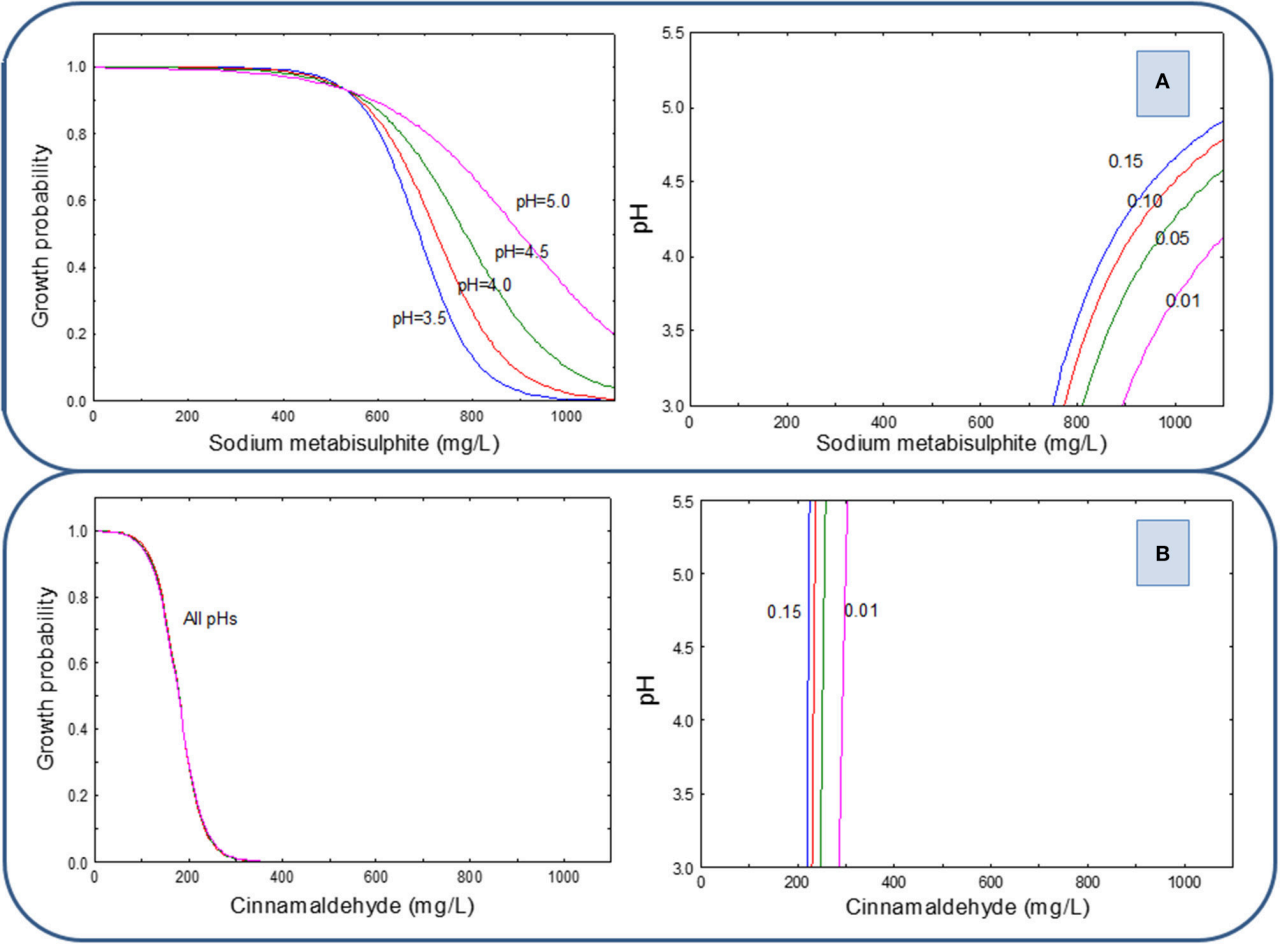
**Growth probability and G/NG interfaces of the yeast cocktail as a function of SM (A) and CIN (B) concentrations in a pH range from 3.5 to 5.0, using PYR as an acidifying agent**.

When the pH correction was achieved with PYR (Figure 4), the concentrations of SM required for yeast inhibition was higher; in fact, the inhibition only begins at concentrations above 500 mg/L and was completely obtained at levels higher than 1000 mg/L at pH values of 3.5 and 4.0. At higher pH values, it would not be possible to reach complete inhibition even with the highest used preservative proportions of SM (Figure 4A left). Then, the experimental region for the G/NG interfaces at *p* = 0.01 is reduced to the region above 900 mg/L SM and pH below 4.0 (Figure 4A right). On the other hand, CIN in the presence of PYR reached full inhibition at concentrations above 300 mg/L solution, regardless of pH value (Figure 4B).

### Logistic models for the *Enterobacteriaceae* cocktail

For this gram-negative bacteria, a total of 410 cases were analyzed, (205 for SM and 205 for CIN), with a distribution of G/NG data of 80/125 for SM and 47/158 for CIN. The probabilistic models for preservatives fit the data satisfactorily, as supported by the statistical tests applied to the model fit (Table S1 in Supplementary Material). The goodness of fit was also assessed by the overall hit (accuracy) to the data used in model development and validation (Table S2 in Supplementary Material), which indicate an almost perfect segregation between G/NG treatments. This way, the specificity was 99 (SM) and 100% (CIN) while the sensitivity was 100 (SM) and 100% (CIN). Furthermore, the predictions obtained for the 150 validation cases (75 for SM and 75 for CIN) also led to 100% high specificity and sensitivity, regardless of preservative assayed. Therefore, the models achieved an adequate segregation between G/NG data and they are appropriate for representing the G/NG *Enterobacteriaceae* interfaces as a function of the levels of SM, CIN, pH, and type of acid.

As in the other microbial groups, the models for logit (p) for *Enterobacteriaceae* population was deduced from the estimated coefficients (Table S5 in Supplementary Material) by applying the same methodology previously described. The following equations were obtained:

$$\begin{array}{l} \text{For SM-HCl}:\text{ logit}(\text{p})\;=-342.118+77.307^{*}\text{pH}+0.426^{*}\\ \;[\text{SM}]-0.102^{*}{\text{pH}}^{*}[\text{SM}]\\ \text{For SM}-\text{PYR}:\text{ logit}(\text{p})=-58.143+13.729^{*}\text{pH}+0.041^{*}\\ \;[\text{SM}]-0.010^{*}{\text{pH}}^{*}[\text{SM}]\\ \text{For CIN}-\text{HCl}:\text{ logit}(\text{p})=-91.283+21.968^{*}\text{pH}+0.410^{*}\\ \;[\text{CIN}]-0.114^{*}{\text{pH}}^{*}[\text{CIN}]\\ \text{For CIN}-\text{PYR}:\text{ logit}(\text{p})=-41.204+9.383^{*}\text{pH}+0.103^{*}\\ \;[\text{CIN}]-0.027^{*}{\text{pH}}^{*}[\text{CIN}] \end{array}$$

**Figure 5:**
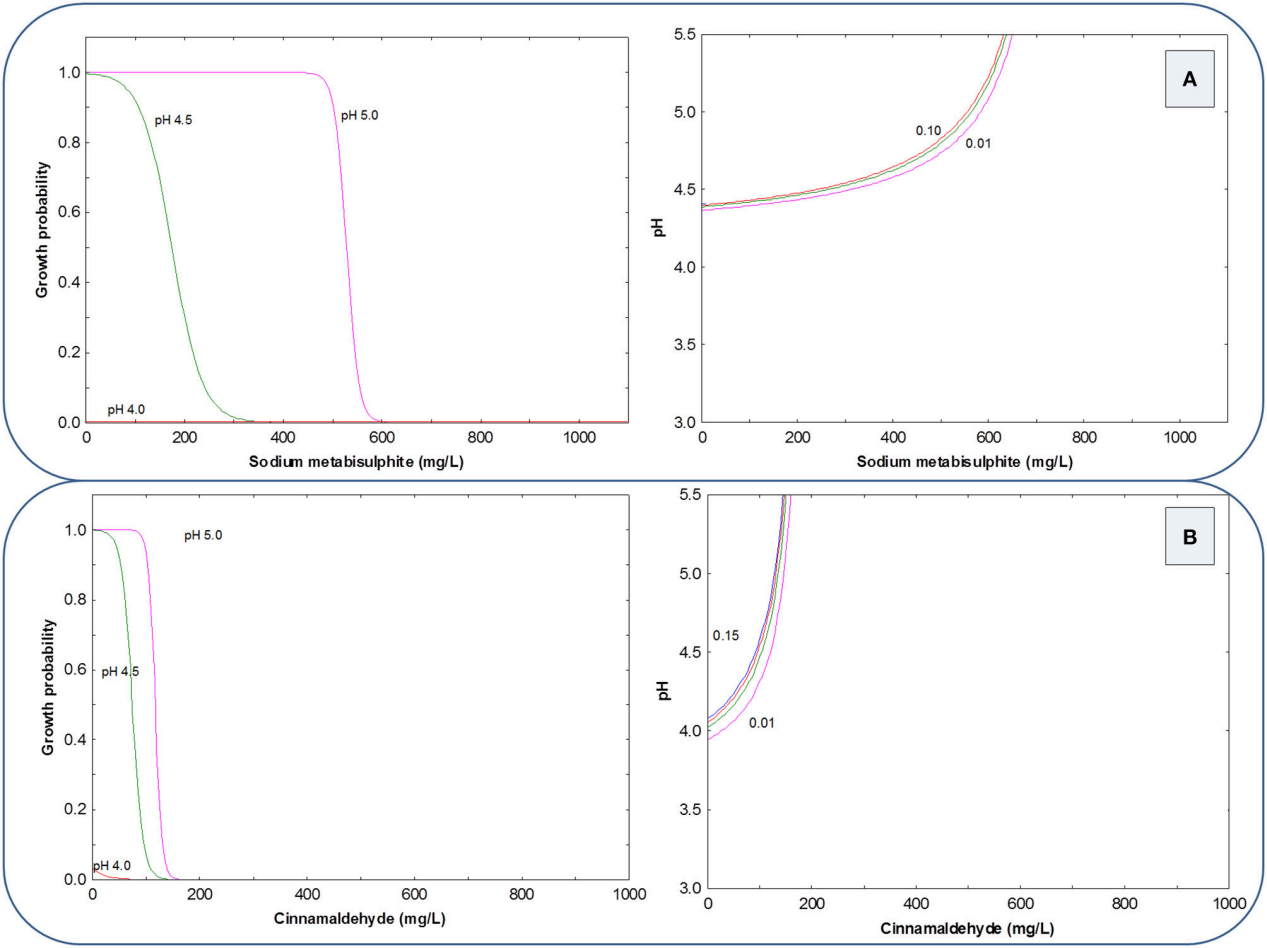
**Growth probability and G/NG interfaces of the *Enterobacteriaceae* cocktail as a function of SM (A) and CIN (B) concentrations in a pH range from 3.5 to 5.0, using HCl as acidifying agent**.

The graphical representation of the growth probability using HCl as acidifying agent shows the great importance that pH had on the growth of this microbial group (Figure 5A left). *Enterobacteriaceae* were not able to grow below pH 4.0 (even in the absence of preservatives) but the increasing levels of pH required higher concentrations of SM (curves shifted to the right). At pH 5.0, it was necessary to add approximately 600 mg/L of SM for the total inhibition of *Enterobacteriaceae* population, while it was necessary the presence of only 350 mg/L SM at pH 4.5 (Figure 5A left). The effect of pH was scarcely noticed in the case of CIN in which the growth probability curves for 4.5, and 5.0 pH values were fairly close, and the total inhibition at both pH values was obtained with lower CIN concentrations, approximately 150 mg/L (Figure 5B left). The G/NG interfaces at different growth probabilities (Figures 5A,B right) confirm that higher concentrations of SM are necessary to control *Enterobacteriaceae* cocktail at higher pHs while the CIN inhibition is less influenced by the pH which interaction was limited to values below 200 mg/L concentration.

**Figure 6:**
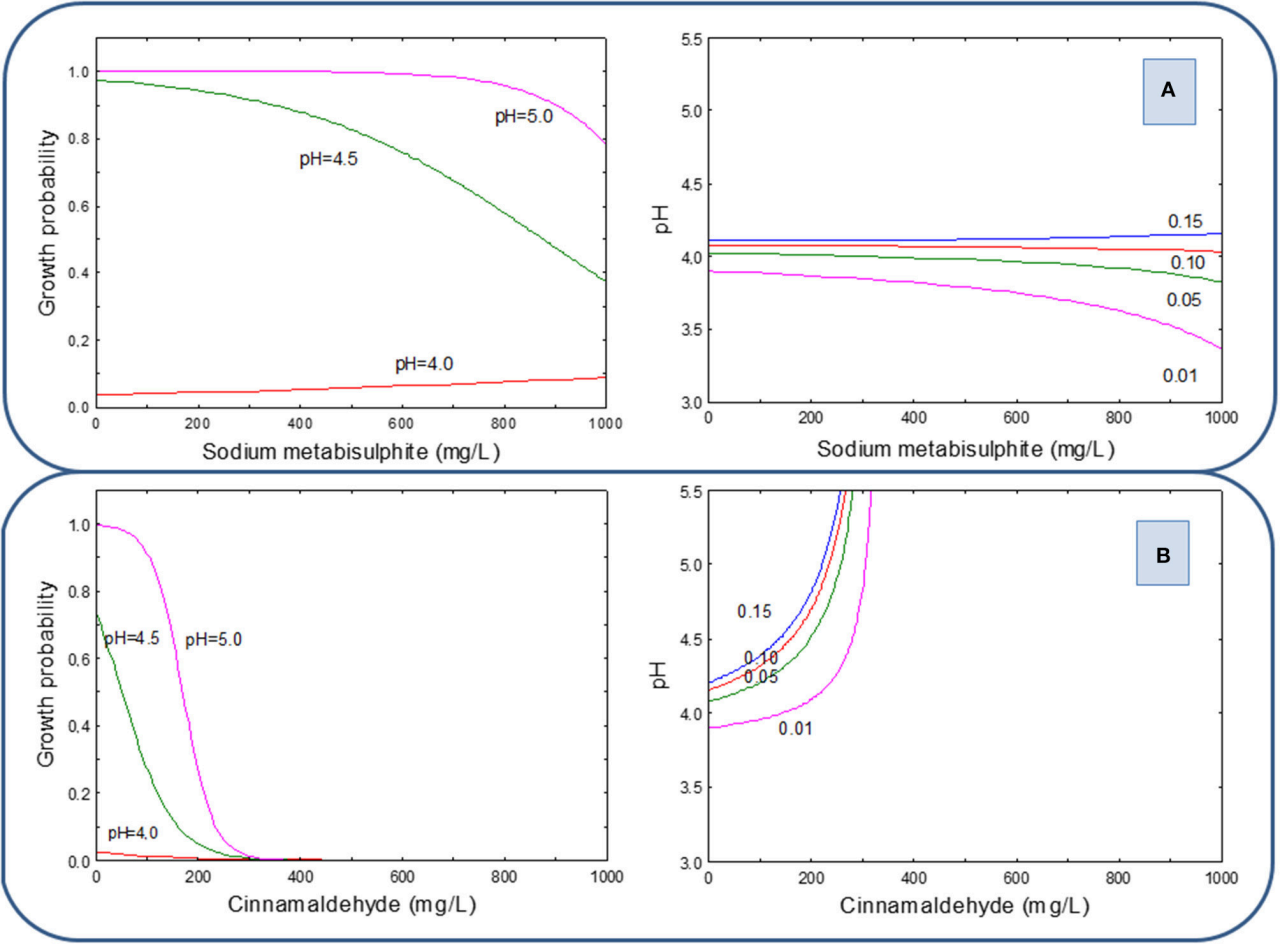
**Growth probability and G/NG interfaces of the ***Enterobacteriaceae*** cocktail as a function of SM (A) and CIN (B) concentrations in a pH range from 3.5 to 5.0, using PYR as an acidifying agent**.

As in the previous microbial groups, the pH correction with PYR caused a marked diminution of the inhibitory effect of SM, which only achieved almost complete inhibition below a pH value of 4.0 and total at 3.5, regardless of the preservative concentration assayed (Figure 6A left). As a result, at pH 4.0, the G/NG interfaces were fairly horizontal with a slight downward curvature (lower pH values) for high SM contents. On the contrary, the effect of PYR on the inhibitory effect CIN was less appreciable and decreased as the preservative concentration increased leading to complete inhibition above approximately 350 mg/L, regardless of the pH values (Figure 6B).

## Discussion

The control of spoilage microorganisms is one of the most important aspects of food preservation and also a prominent issue in the stability of table olive packaging. Many of the food preservatives habitually used by industry for this purpose are weak acids, such as sorbic, benzoic, propionic, acetic, and sulphites (Piper, 2011). Weak acids are widely used in low-pH foods, where its inhibitory power increases. Therefore, they could have direct application in table olive packaging, albeit the information on their effects on the main microbial groups found in table olives environment is scarce. This survey tries to elucidate the applicability of SM and CIN as preservatives to control the growth of table olive related microorganisms (bacteria and fungi), as an alternative to classic sorbic, and benzoic acids. To this aim, three different cocktails (LAB, yeast, and *Enterobacteriaceae*) that mimicked the microbiota in real olive packages were assayed. The use of a microbial cocktail rather than individual species is a convenient and faster way of checking the overall effects that inhibitory compounds could have against a specific microbial group. This way, the G/NG boundaries will be obtained for the most resistant strain of the cocktail. This strategy has been successfully used in food microbiology to estimate the overall response of microorganisms as a function of storage conditions or preservatives (Arroyo-López et al., 2012; Leong et al., 2014).

Ratkowsky and Ross (1995) were the first researchers modeling the bacterial G/NG interfaces. Years later, Presser et al. (1998) and Lanciotti et al. (2001) used the visible increase of turbidity to deduce the growth limits of *E. coli, Bacillus cereus, Staphylococcus aureus*, and *Salmonella enteritis*. The application of the logistic/probabilistic models (based on OD) to determine the G/NG boundaries of both bacteria and yeasts is rather habitual in predictive microbiology. Among the environmental variables included in the model, temperature, pH, *a*_*w*_, organic acids, or preservatives are usual (Boziaris et al., 2006; Valero et al., 2010; Arroyo-López et al., 2012; Astoreca et al., 2012; Tabanelli et al., 2014). That is, determination of the G/NG interfaces of spoilage and pathogen microorganisms, based on OD, at selected growth probabilities levels has become a standard practice for establishing conditions to avoid economic losses and outbreak food illness, respectively. To our knowledge, this is the first probabilistic model built for SM and CIN using the main microbial groups found in table olive environment as targets.

SO_2_ is widely used in both wine and food industries for its antioxidant and antimicrobial properties. Once dissolved in water, SO_2_ exists in equilibrium between molecular SO_2_, bisulphite, and sulphite forms. This equilibrium is dependent on pH of the medium, with the bisulphite anion being the dominant form under olive packaging conditions (pH between 3.5 and 4.0). Apparently, only molecular SO_2_ exerts an antimicrobial action, and its concentration in food depends on of many factors such as pH and temperature (Fugelsang and Edwards, 2007). Thus, its inhibitory power has similar behavior than other weak organic acids such as sorbic and benzoic acids, being pH dependent. In the present study, SM was more useful to control bacteria than yeasts growth. Our results contrast with those obtained by Rojo-Bezares et al. (2007), who found a higher resistance of LAB (12.8 mg/L) than yeasts (1.6 mg/L) against the inhibitory effects of potassium metabisulphite in laboratory media at pH 3.5. On the contrary, the results obtained from this work are in agreement with Chang et al. (1997) who reported that 100 mg/L of sulphite concentration inhibited the growth of *Lactobacillus fermentun* and *Lactobacillus casei*, but it was necessary up to 500 mg/L to inhibit *S. cerevisiae* growth. In line with these results, Taboada-Rodríguez et al. (2013) found that SM did not show any fungicide effect when used as a preservative for dealcoholized red wine, using *Rhodotorula mucillaginosa* and *S. cerevisiae* as target organisms. The only probabilistic model for SO_2_ as a function of pH was recently developed by Sturm et al. (2014) for *Dekkera bruxellensis*. These authors found that the effect of SO_2_ on yeast G/NG boundaries was considerably affected by the pH of the medium, being necessary lower SO_2_ levels as pH decreased. According to the General Standard for Food Additives (CODEX Stand 192-1995, rev 2014) the use of metabisulphite is permitted for the products included in the Food Category num. 04.2.2.3 (which includes table olives). The recently issued CODEX Standard for Table Olives (CODEX Stan 66-1891 rev 2013) also refers to General Standard in the section related to food additives. However, according to Directive (CE) N° 1129/2011 [European Parliament and Council (EU), 2011], which follows a similar scheme and criterion that the Food Additive Standards issued by the CODEX, the metabisulphite, although allowed for products in the food category num. 04.2.2 (which includes olives), is explicitly excluded for table olives, and yellow peppers in brine. Apparently, the re-introduction of this additive in the Standard issued by the CODEX (rev. 2014) has not implied the subsequent rectification in the European Directive, despite the diverse modifications it has suffered in the last few years. However, metabisulphite was traditionally used in table olives until its temporary removal from the Food Additive Standards issued by the CODEX, which also caused its elimination from the Directive (CE) N° 1129/2011 [European Parliament and Council (EU), 2011] and the Trade Standard for Table Olive (IOOC, 2004). However, after the re-inclusion of the metabisulphite use in CODEX Stan 192-1995, rev 2014) neither of these legislative organisms has updated the metabisulphite status. Nowadays, the discrepancies between the EU legislation and CODEX may lead to disputes and insecurity in the international table olive trade. Thus, studies on the inhibitory effects on table olive related microorganisms are necessary to assist legislators on the homogenization of Standards. Besides, its use in table olives would be convenient due to its antioxidant (browning prevention) and inhibitory effects on the microbial populations (Arroyo-López et al., 2008; Echevarria et al., 2010). Furthermore, SM may also remain as a result of its use as antioxidant during postharvest treatments (Segovia-Bravo et al., 2010) and this carry over effect should also be considered. In a previous study with table olive related microorganisms, this compound had a moderate inhibitory effect in laboratory medium against yeast (MIC value approximately 770 ppm) and especially against LAB cocktails (MIC value 50 ppm; Romero-Gil et al., 2016). However, a concentration of 1500 ppm was not enough to inhibit LAB and yeast populations in real olive fermentations for 2 months, albeit showed a higher inhibitory effect than ascorbic acid (Echevarria et al., 2010). Taking into consideration all these studies, probably the metabisulphite levels necessary to inhibit LAB growth could be compatible with the usual olive packaging procedures. On the contrary, the higher doses necessary to control yeast growth might cause allergic reactions, and headache especially in sensitive persons to this preservative. In the specific case of table olives, its residual current level should be below 100 mg/kg flesh (expressed as sulfur dioxide) as established in the CODEX Stan 192-1995. At this level, any possible health effect would be markedly reduced for most consumers. In wines, the maximum allowable limits for the addition of SO_2_ by the OIV is from 150 to 300 mg/L of total SO_2_ (OIV, 1998).

The pH of the medium influenced less the inhibitory effect of CIN compared to SM, which is a considerable advantage compared to other weak organic acids, and the own sulphites. Yeasts and *Enterobacteriaceae* were the microbial groups more inhibited by this preservative, and levels of approximately 150 mg/L were enough to prevent their growth. Data obtained in a previous work demonstrated that this organic compound was effective to control table olive microorganisms, but its effect was microbial group dependent, with a higher inhibitory effect against yeast (125 ppm) than on LAB (1060 ppm; Romero-Gil et al., 2016). Its application in olive packaging should be accompanied by sensorial evaluation to determine the influence of the required inhibitory levels on the flavor of the final products due to the characteristic smell of this compound to cinnamon. Recently, CIN was applied to stabilize acidified cucumbers that were adequately preserved free of yeasts (Pérez-Díaz, 2011). Considering the efficient inhibition of yeast in cucumbers, testing CIN against the microorganisms present in table olives may be interesting, especially for the development of new flavored table olives. This compound is obtained from the cinnamon bark. The mechanism of the bactericidal action of CIN against *Listeria monocytogenes*, possible inhibition of glucose uptake, and utilization and effects on membrane permeability, was suggested by Gill and Holley (2004). This compound had both antimicrobial and antioxidant activities when applied to meat, thus, preventing microbial spoilage, and lipid oxidation (Naveena et al., 2013). Carvacrol and CIN were associated with an easier thermal destruction of *E. coli* O157:H7 (Juneja and Friedman, 2008) in raw ground meat: the addition of increasing levels of carvacrol or CIN (range 0.5–1.0%) significantly increased the sensitivity of the microorganism to heat. However, this property would not be of interest for table olives since the application of thermal treatments to the seasoned products would sensibly affect their characteristic flavors. CIN has been reported to show a potential inhibitory effect on methicillin-resistant *S. aureus* biofilm-related to infections (Jia et al., 2011). Recently, CIN has been suggested as a useful compound for the control of *E. coli* at refrigeration temperature (Visvalingam and Holley, 2012). The use of encapsulation is currently observed as an interesting challenge for the application of not only CIN but also other essential (or not) oils (Sagiri et al., 2016).

PYR has a low pK_a_ value (2.39), a circumstance that allows its use for acidification purposes in table olives. However, a loss of its inhibitory power was observed for SM and CIN preservatives in the presence of this acidifying agent. PYR was useful to control both LAB and yeast growth when applied individually at concentrations around 3200 mg/L (Romero-Gil et al., 2016). This compound was first patented for its preservative properties by Ernst et al. (1979) to stabilize high moisture food products without refrigeration. PYR (and acetaldehyde)-bound sulfur dioxide produced inhibition against wine LAB at a concentration of 5 ppm, albeit the LAB finally degraded such compounds, suggesting that sulfur dioxide-bound PYR could have a bacteriostatic effect rather than a bactericidal action (Wells and Osborne, 2011).

## Conclusions

The *in silico* models obtained have shown that SM and CIN preservatives were very efficient to control the growth of the main microbial groups found in table olive environment and that HCl is the best acidifying agent. The response of microorganisms as a function of preservative concentration was also different, being the inhibitory effects of SM higher for LAB than for yeast at the same levels of pH, while the opposite behavior was noticed for CIN. The *Enterobacteriaceae* group was markedly affected by the pH of the medium, and they were not able to grow at a pH below 4.0 even in the absence of preservatives. Further verification of these results in synthetic brines as well as validation in real conditions (table olive packaging) are now needed.

## Author contributions

VR performed the experimental work. AG and FA designed the work, analyzed the results and written the paper.

### Conflict of interest statement

The authors declare that the research was conducted in the absence of any commercial or financial relationships that could be construed as a potential conflict of interest.

## Footnotes

^1^CODEX Stand, 66-1982. (2013). General Standard for Table Olives. *Rev* 2013. pp 9.

^2^CODEX Stand, 192-1995. (2014). General Standard for Food Additives. Rev 2014. *pp* 264–265.
