# Supplementary material for: In silico Logistic Model for Table Olive Related Microorganisms As a Function of Sodium Metabisulphite, Cinnamaldehyde, pH, and Type of Acidifying Agent
Source: Front Microbiol. 2016 Aug 31;7:1370. doi: 10.3389/fmicb.2016.01370 (PMC5005353; doi:10.3389/fmicb.2016.01370)
Supplement: Supplementary file 1 [file Table1.DOCX]

**Table S1.** Fit parameters for the logistic models (G/NG interfaces) according to microbial groups and type of preservative assayed.

| Treatment | Fit parameters | | | | | | | | | | |
| --- | --- | --- | --- | --- | --- | --- | --- | --- | --- | --- | --- |
|  | R^2^ | | | -2log(likehood) | | Score | | Wald | | Hosmer-Lemeshow | |
|  | McFadden | Nagelkerke | Cox &Snell | Chi^2^ | p>Chi^2^ | Chi^2^ | p>Chi^2^ | Chi^2^ | p>Chi^2^ | Chi^2^ | p>Chi^2^ |
| **LAB** |  |  |  |  |  |  |  |  |  |  |  |
| SM | 0.817 | 0.671 | 0.902 | 324 (7) | <0.000 | 174 (7) | <0.000 | 44 (7) | <0.000 | 2.66 (8) | 0.954 |
| CIN | 0.917 | 0.617 | 0.951 | 256 (7) | <0.000 | 193 (7) | <0.000 | 40 (7) | <0.000 | 1.46 (8) | 0.993 |
| **Yeasts** |  |  |  |  |  |  |  |  |  |  |  |
| SM | 0.921 | 0.586 | 0.951 | 257 (7) | <0.000 | 219 (7) | <0.000 | 33 (7) | <0.000 | 2.55 (8) | 0.959 |
| CIN | 0.864 | 0.697 | 0.930 | 348 (7) | <0.000 | 167 (7) | <0.000 | 33 (7) | <0.000 | 1.72 (8) | 0.988 |
| ***Enterobacteriaceae*** |  |  |  |  |  |  |  |  |  |  |  |
| SM | 0.926 | 0.710 | 0.963 | 254 (7) | <0.000 | 160 (7) | <0.000 | 24 (7) | <0.000 | 2.15 (8) | 0.975 |
| CIN | 0.882 | 0.613 | 0.930 | 194 (7) | <0.000 | 124 (7) | <0.000 | 22 (7) | <0.000 | 3.99 (8) | 0.858 |

Note: SM, sodium metabisulphite. CIN, cinnamaldehyde

**Table S2.** Classification of samples (G/NG) deduced by the logistic regression model for the diverse preservatives assayed for both the samples used for obtaining the model and for those used as validation.

|  | **Model building samples** | | | | | **Validation** | | | |
| --- | --- | --- | --- | --- | --- | --- | --- | --- | --- |
| ***Preservative** | **From/to** | **0** | **1** | **Total** | **% correct** | **0** | **1** | **Total** | **% correct** |
| **LAB** | | | | | | | | |  |
| SM | 0 | 116 | 7 | 123 | 94.31 | 37 | 2 | 39 | 94.87 |
|  | 1 | 4 | 165 | 169 | 97.63 | 4 | 57 | 61 | 93.44 |
|  | Total | 120 | 172 | 292 | 96.23 | 41 | 57 | 100 | 94.00 |
| CIN | 0 | 58 | 0 | 58 | 100.00 | 17 | 3 | 20 | 85.00 |
|  | 1 | 1 | 208 | 209 | 99.52 | 1 | 104 | 105 | 99.05 |
|  | Total | 59 | 208 | 267 | 99.63 | 18 | 107 | 125 | 95.80 |
| **Yeasts** | | | | | | | | | |
| SM | 0 | 54 | 0 | 54 | 100.00 | 14 | 1 | 15 | 93.33 |
|  | 1 | 2 | 236 | 238 | 99.16 | 0 | 85 | 85 | 100.00 |
|  | Total | 56 | 236 | 292 | 99.32 | 14 | 86 | 100 | 99.00 |
| CIN | 0 | 131 | 5 | 136 | 96.32 | 47 | 1 | 48 | 97.92 |
|  | 1 | 3 | 153 | 156 | 98.08 | 1 | 51 | 52 | 98.08 |
|  | Total | 134 | 158 | 292 | 97.26 | 48 | 52 | 100 | 98.00 |
| ***Enterobacteriaceae*** | | | | | | | | | |
| SM | 0 | 125 | 0 | 125 | 100.00 | 45 | 0 | 45 | 100.00 |
|  | 1 | 1 | 79 | 80 | 98.75 | 0 | 30 | 30 | 100.00 |
|  | Total | 126 | 79 | 205 | 99.51 | 45 | 30 | 75 | 100.00 |
| CIN | 0 | 155 | 3 | 158 | 98.10 | 55 | 0 | 55 | 100.00 |
|  | 1 | 0 | 47 | 47 | 100.00 | 0 | 20 | 20 | 100.00 |
|  | Total | 155 | 50 | 205 | 98.54 | 55 | 20 | 75 | 100.00 |

*SM, sodium metabisulphite. CIN, cinnamaldehyde

**Table S3**. Estimated coefficients of the probabilistic model for lactic acid bacteria as a function of preservatives, pH, and type of acid.

| **Preservative*** | **Coefficient** | **Value** | **Standard error** | **Wald Chi^2^** | **Pr>Chi^2^** | **Wald Low Limit (95%)** | **Wald High Limit (95%)** | **Odds ratio** |
| --- | --- | --- | --- | --- | --- | --- | --- | --- |
| SM | Intercept | -9.983 | 3.660 | 7.439 | 0.006 | -17.157 | -2.809 | - |
|  | pH | 3.024 | 0.931 | 10.559 | 0.001 | 1.200 | 4.848 | 20.572 |
|  | [SM] | -0.077 | 0.046 | 2.795 | 0.095 | -0.167 | 0.013 | 0.926 |
|  | A-PYR  pH*[SM] | -54.896  0.011 | 18.344  0.010 | 8.956  1.317 | 0.003  0.251 | -90.849  -0.008 | -18.943  0.030 | 0.000  1.011 |
| CIN | pH*A-PYR  [SM]*A-PYR  pH*[SM]*A-PYR  Intercept | 14.264  0.101  -0.018  24.325 | 4.875  0.054  0.012  12.797 | 8.559  3.574  2.107  3.613 | 0.003  0.059  0.147  0.057 | 4.708  -0.004  -0.041  -0.757 | 23.819  0.206  0.006  49.407 | 1.6·10^6^  1.107  0.983  - |
|  | pH | -4.305 | 2.768 | 2.419 | 0.120 | -9.730 | 1.120 | 0.013 |
|  | [CIN] | -0.125 | 0.049 | 6.586 | 0.010 | -0.220 | -0.029 | 0.883 |
|  | A-PYR  pH*[CIN] | -83.519  0.029 | 21.185  0.012 | 15.543  5.996 | <0.0001  0.014 | -125.041  0.006 | -41.998  0.052 | 0.000  1.029 |
|  | pH*A-PYR  [CIN]*A-PYR  pH*[CIN]*A-PYR | 20.504  0.146  -0.003 | 5.482  0.054  0.013 | 13.987  7.287  7.372 | 0.000  0.007  0.007 | 9.758  0.040  -0.061 | 31.249  0.249  -0.010 | 8.0·10^8^  1.115  0.965 |

*SM, sodium metabisulfite; CIN, cinnamaldehyde; A, type of acid used for pH correction; PYR, pyruvic acid. The reference level for the type of acid was HCl. The G/NG interface equation for HCl is obtained by summing all the terms without A-PYR, while for PYR is formed by summing to the previous ones those terms which include A-PYR (without this expression) and simplifying the expression.

**Table S4**. Estimated coefficients of the probabilistic model for yeasts as a function of preservatives, pH, and type of acid.

| **Preservative*** | **Coefficient** | **Value** | **Standard error** | **Wald Chi^2^** | **Pr>Chi^2^** | **Wald Low Limit (95%)** | **Wald High Limit (95%)** | **Odds ratio** |
| --- | --- | --- | --- | --- | --- | --- | --- | --- |
| SM | Intercept | -26.199 | 15.597 | 2.822 | 0.093 | -56.769 | 4.371 | - |
|  | pH | 8.915 | 4.375 | 4.152 | 0.042 | 0.340 | 17.491 | 7446 |
|  | [SM] | -0.063 | 0.025 | 6.250 | 0.012 | -0.133 | -0.014 | 0.939 |
|  | A-PYR  pH*[SM] | 49.649  0.009 | 22.940  0.005 | 4.684  3.055 | 0.030  0.080 | 4.684  -0.001 | 94.607  0.020 | 3.6·10^20^  1.009 |
| CIN | pH*A-PYR  [SM]*A-PYR  pH*[SM]*A-PYR | -12.332  0.024  -0.003 | 5.703  0.035  0.007 | 4.675  0.477  0.161 | 0.031  0.490  0.688 | -23.510  -0.044  -0.017 | -1.153  0.092  0.011 | 0.000  1.024  0.997 |
|  | Intercept  pH | 4.507  0.431 | 12.938  3.055 | 0.121  0.020 | 0.728  0.888 | -20.851  -5.557 | 29.865  6.419 | -  1.539 |
|  | [CIN] | -0.062 | 0.139 | 0.196 | 0.658 | -0.334 | 0.211 | 0.940 |
|  | A-PYR  pH*[CIN] | 4.623  0.000 | 18.272  0.033 | 0.064  0.000 | 0.800  0.989 | -31.188  -0.065 | 40.435  0.064 | 1.011  1.000 |
|  | pH*A-PYR  [CIN]*A-PYR  pH*[CIN]*A-PYR | -0.927  0.011  0.003 | 4.278  0.156  0.037 | 0.047  0.005  0.007 | 0.828  0.944  0.933 | -9.311  -0.295  -0.069 | 7.457  0.317  0.075 | 0.396  1.011  1.003 |

*SM, sodium metabisulfite; CIN, cinnamaldehyde; A, type of acid used for pH correction; PYR, pyruvic acid. The reference level for the type of acid was HCl. The G/NG interface equation for HCl is obtained by summing all the terms without A-PYR, while for PYR is formed by summing to the previous ones those terms which include A-PYR (without this expression) and simplifying the expression.

**Table S5**. Estimated coefficients of the probabilistic model for *Enterobacteriaceae* as a function of preservatives, pH, and type of acid.

| **Preservative*** | **Coefficient** | **Value** | **Standard error** | **Wald Chi^2^** | **Pr>Chi^2^** | **Wald Low Limit (95%)** | **Wald High Limit (95%)** | **Odds ratio** |
| --- | --- | --- | --- | --- | --- | --- | --- | --- |
| SM | Intercept | -342.118 | 127.790 | 7.167 | 0.007 | -592.582 | -91.654 | - |
|  | pH | 77.307 | 28.790 | 7.211 | 0.007 | 20.881 | 133.734 | 3.8·10^33^ |
|  | [SM] | 0.426 | 0.164 | 6.703 | 0.010 | 0.103 | 0.748 | 1.530 |
|  | A-PYR  pH*[SM] | 283.974  -0.102 | 129.405  0.039 | 4.816  6.747 | 0.028  0.009 | 30.345  -0.179 | 537.604  -0.025 | 2.1·10^123^  0.900 |
| CIN | pH*A-PYR  [SM]*A-PYR  pH*[SM]*A-PYR | -63.578  -0.384  0.092 | 29.183  0.166  0.040 | 4.746  5.342  5.360 | 0.029  0.021  0.021 | -120.776  -0.710  0.014 | -6.381  -0.058  0.169 | 0.000  0.681  1.100 |
|  | Intercept  pH | -91.283  21.969 | 28.176  6.929 | 10.496  10.053 | 0.001  0.002 | -146.506  8.389 | -36.059  35.548 | -  5.6·10^9^ |
|  | [CIN] | 0.410 | 0.165 | 6.185 | 0.013 | 0.087 | 0.733 | 1.510 |
|  | A-PYR  pH*[CIN] | 50.078  -0.114 | 32.433  0.046 | 2.384  6.066 | 0.123  0.014 | -13.490  -0.204 | 113.646  -0.023 | 5.6·10^21^  0.893 |
|  | pH*A-PYR  [CIN]*A-PYR  pH*[CIN]*A-PYR | -12.585  -0.307  0.086 | 7.810  0.170  0.047 | 2.597  3.270  3.317 | 0.107  0.071  0.069 | -27.892  -0.641  -0.007 | 2.722  0.026  0.179 | 0.000  0.735  1.090 |

*SM, sodium metabisulfite; CIN, cinnamaldehyde; A, type of acid used for pH correction; PYR, pyruvic acid. The reference level for the type of acid was HCl. The G/NG interface equation for HCl is obtained by summing all the terms without A-PYR, while for PYR is formed by summing to the previous ones those terms which include A-PYR (without this expression) and simplifying the expression.
